# Liver transplantation in rare late‐onset ornithine transcarbamylase deficiency with central nervous system injury: A case report and review of the literature

**DOI:** 10.1002/brb3.2765

**Published:** 2022-09-20

**Authors:** Xin Jin, Xinchen Zeng, Dong Zhao, Nan Jiang

**Affiliations:** ^1^ Division of Liver Surgery and Organ Transplantation Center, Shenzhen Third People's Hospital Second Affiliated Hospital of Southern University of Science and Technology, National Clinical Research Center for Infectious Disease Shenzhen China

**Keywords:** central nervous system injury, hydrocephalus, hyperammonemia, liver transplantation, ornithine transcarbamylase deficiency

## Abstract

**Background:**

Ornithine transcarbamylase deficiency (OTCD) is a genetic metabolic disease. Its clinical manifestations are mainly central nervous system dysfunction caused by high blood ammonia. Late‐onset OTCD combined with central nervous system injury has a poor therapeutic response, which is one of the main factors affecting the prognosis and quality of life of patients. liver transplantation (LT) has gradually become a radical treatment for OTCD, which has achieved good results. However, there is no consensus on the timing of LT and problems of nervous system damage and repair.

**Methods:**

We report the development of late‐onset OTCD with central nervous system injury in an 11‐year‐old child who received liver transplantation at our transplant center. His first symptoms were nonprojectile vomiting, followed by irritability and disturbance of consciousness, after which the disease progressed rapidly and finally resulted in a coma. After liver transplantation, the child's consciousness returned to normal, muscle strength of the limbs gradually recovered from grade 0 to grade 4, and muscle tone gradually recovered from grade 4 to grade 1, suggesting that the motor nerves had gradually recovered. However, the child is currently mentally retarded, and the language center has not yet fully recovered.At the same time, we made a literature review of OTCD.

**Conclusion:**

For OTCD patients with central nervous system injury, liver transplantation can fundamentally solve the problem of ammonia metabolism in the liver and avoids further damage to the central nervous system caused by hyperammonemia. At the same time, children's nervous systems are in the developmental stage when neuroplasticity is greatest. If liver transplantation is performed as soon as possible, nerve repair is still possible.

## Graphical Abstract

1. Ornithine transcarbamylase deficiency (OTCD) is a kind of genetic metabolic disease. Its clinical manifestations are mainly central nervous system dysfunction caused by high blood ammonia. 2. Liver transplantation can fundamentally solve the problem of ammonia metabolism in the liver and avoid further damage to the central nervous system caused by hyperammonemia.

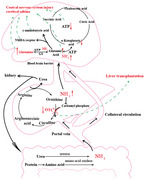

## 1 INTRODUCTION

Urea cycle disorder (UCD) is a rare genetic disorder, mainly caused by a deficiency of six key enzymes or two transporters that remove ammonia from the body (Matsumoto et al., 2019). Among them, ornithine transcarbamylase (OTC) is a mitochondrial enzyme encoded by the OTC gene located on Xp21.1 (Lindgren et al., 1984), which can catalyze the formation of citrulline from carbamyl phosphate and ornithine. The mutation of the OTC gene will lead to X‐linked ornithine transcarbamylase deficiency (OTCD), also known as hyperammonemia type II. According to the literature, the incidence rate of OTCD is from 1/60,000 to 1/17,000, and OTCD is the most common disease related to UCD, accounting for 1/2 to 2/3 of cases (Brassier et al., 2015; Kido et al., 2012; Summar et al., 2013). The onset time of this disease is wide, but it occurs mostly in newborns and infants, and is less likely in children and adults. According to the onset time of OTCD, cases can be divided into neonatal OTCD (≤ 30 days after birth) and late‐onset OTCD (> 30 days after birth). The disease starts insidiously and some patients show normal intelligence development before the onset of the disease. The disease can be induced by high protein, infection, drugs, and other factors. The course of the disease is intermittent and progresses slowly. The main clinical manifestations are central nervous system dysfunction caused by high blood ammonia, such as irritability, intellectual disorders, epilepsy, sleepiness, coma, developmental retardation, dysplasia, vomiting, and psychological and behavioral problems (Savy et al., 2018). Persistent hyperammonemia can cause irreversible and severe damage to the central nervous system of patients, and even result in serious neurological and mental damage. This is also the fundamental cause of death in OTCD patients (Maestri et al., 1991; Tuchman, 1992). OTCD is easy to misdiagnose due to the lack of specific clinical manifestations and limitations in specific clinical detection methods. In recent years, liver transplantation (LT) has gradually become a radical treatment for OTCD, which has achieved good results (Hasegawa et al., 1995; Leonard & McKiernan, 2004; McBride et al., 2004; Morioka et al., 2005; Summar et al., 2008; Whitington et al., 1998; Wraith, 2001). However, there is no consensus on the timing of LT. Some studies have shown that damage to the nervous system caused by hyperammonemia is irreversible. Even after LT, the function of the nervous system that is damaged cannot be restored to normal in some cases (Busuttil et al., 1998; Wakiya et al., 2011; Wraith, 2001). Wakiya et al. (2011) suggested that surgery should be performed on patients in a stable metabolic state without serious nervous system injury. In this article, we report the clinical characteristics and treatment experience of a child with late‐onset OTCD with central nervous system injury treated by LT and review the relevant literature to provide a clinical reference.

## 2 CASE PRESENTATION

The patient, an 11‐year‐old child, was admitted to the hospital for “disorders of consciousness for more than 1 month” (March 12, 2021). More than 1 month previously, the child had unexplained headaches, which were not severe but tolerable, accompanied by nonprojectile vomiting. The vomit contained gastric contents, with a volume of about 100 ml, and occurred 10−12 times per day. At the same time, the child experienced dizziness but had neither blurred vision nor fever or chills. The response to oral administration of domperidone and ibuprofen was poor. One day after admission, the child showed signs of consciousness disturbance and irritability, and immediately went to a local hospital for treatment, including volume resuscitation and mannitol to reduce intracranial hypertension. The child repeatedly became agitated and was sedated many times. The blood ammonia level was more than 500 nmol/L, and the patient was treated to reduce blood ammonia. The next day, the child developed a coma with spontaneous respiration, with a blood pressure of 134/78 mmHg, and no obvious tendon hyperreflexia. Relevant examinations were completed and showed blood ammonia 462 μmol/L, alanine aminotransferase 73 U/L, prothrombin time 23 s, lactic acid 4.47 mmol/L, normal blood lipids, ferritin and ceruloplasmin, and normal anion gap. Blood tandem mass spectrometry showed that the citrulline level was normal, but uracil and lactate acid were increased. Urine gas chromatography‐mass spectrometry showed that lactate acid and uracil were significantly increased and pyruvate was increased. Both of these measurements indicated OTCD. Gene screening revealed OTCD (C.119 [exon 2] G > A mutation). Cranial MRI was consistent with cerebral manifestations of hyperammonemia and showed cerebral edema and no hydrocephalus. Electroencephalogram (EEG) showed that there was no obvious dominant rhythm in the bilateral occipital region. During the waking period, diffuse δ activity was close to persistent release and was slightly more pronounced in the anterior head. During sleep, a pattern of high and low wave amplitude alternated periodically. Local hospital treatments included hemofiltration, reducing ammonia, defecation, inhibition of ammonia production by intestinal bacteria, inhibition of proteolysis, anti‐epilepsy medication, improvement of coagulation function, and other treatment measures. Meanwhile, anti‐infection treatment, mannitol to reduce intracranial hypertension, nutritional supplementation, and probiotics were also given. After active treatment, the patient's condition stabilized, and liver function, blood coagulation function, and blood ammonia returned to normal. Due to a continuous coma, the patient was treated with hyperbaric oxygen twice and then was transferred to our hospital.

**FIGURE 1:**
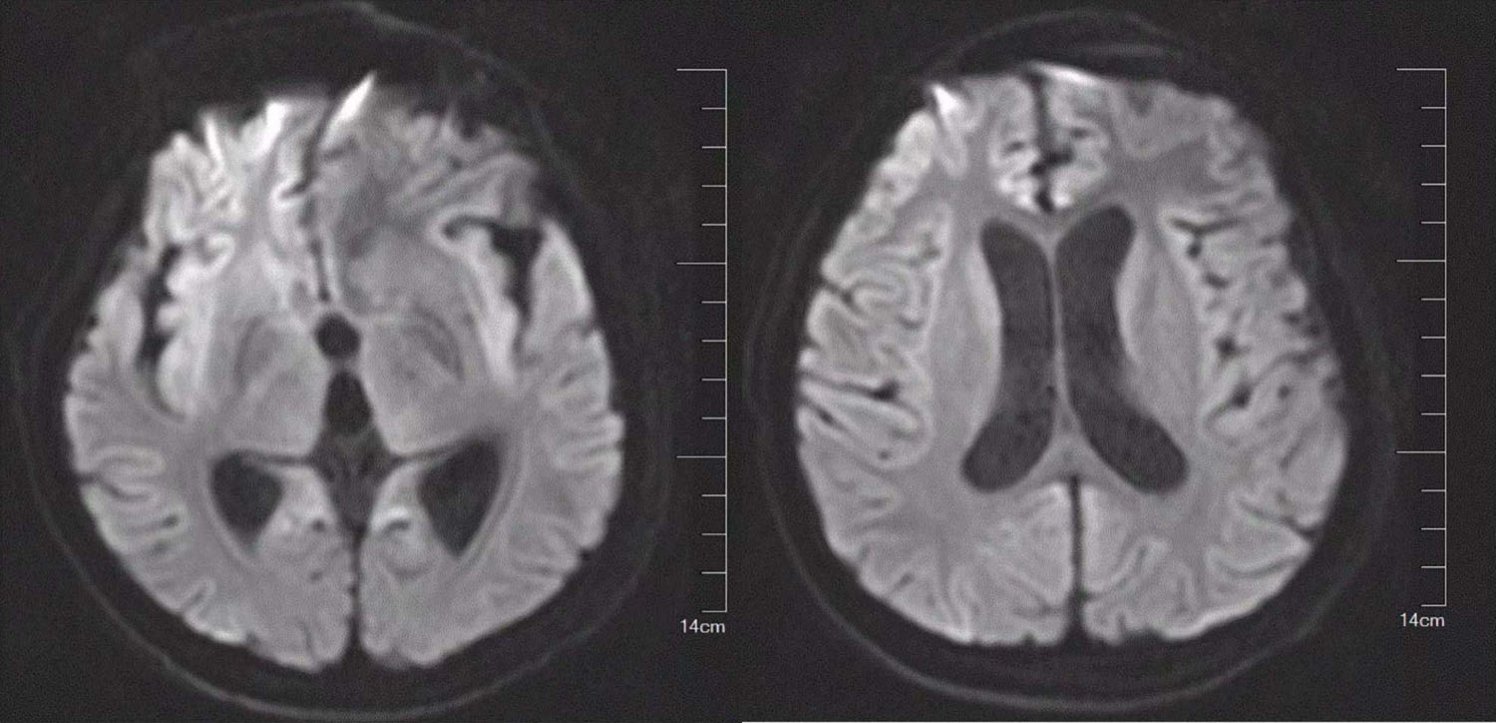
Preoperative cranial MRI

Upon entering our hospital, the child was in a shallow coma state, and the Glasgow Coma Score (GCS) was 3 points. The skin and sclera of the whole body were not yellow and the eyelids could not be closed. The neck was stiff, and fingers and toes were convulsed intermittently with ankylosis. In addition, abdominal reflexes and cremasteric reflexes could not be elicited. Bilateral ankle jerks and knee jerks were elicited, bilateral Babinski reflex and Kernig's sign were negative, and flapping tremor was negative. The muscle strength of the four limbs was grade 0, the muscle tone of both upper limbs was grade 3, and the muscle tone of both lower limbs was grade 1+. There were no other abnormal signs. The child had normal growth and development, and denied a family history of genetic diseases. Routine blood, blood ammonia, liver and kidney function, and coagulation function were normal. Cranial enhanced MRI showed multiple abnormal signal shadows in the right frontal lobe, parietal lobe and lateral fissure, and hydrocephalus (Figure 1). EEG showed no dominant rhythm in the occipital region, and the whole conductor was continuously distributed with low‐to‐medium‐amplitude δ activity mixed with θ waves. Irregular sharp spike waves and sharp spike slow waves were occasionally seen in the frontal area during sleep. Sleep background disorder and sleep cycle could not be distinguished. During the monitoring process, the right lower limb trembled involuntarily, but there was no obvious abnormal change in EEG during the same period. After multidisciplinary discussions, the child met the indications for LT, but the patient had nervous system damage, which indicated that damaged nervous system function might not be recovered after the operation. After a detailed explanation of the child's condition, surgical risks, and postoperative complications with family members, the family members expressed their understanding and strongly desired LT. Allogeneic orthotopic LT (modified piggyback) was performed under general anesthesia (March 18, 2021). The operation was successful and the patient was transferred to the intensive care unit.

On the fifth day after the operation, the child regained consciousness from the coma. The muscle strength of the four limbs was grade 0, while the muscle tone of both upper limbs increased from grade 3 to grade 4, and the muscle tone of both lower limbs increased from grade 1+ to grade 4. GCS score was not obtained due to the high muscle tone of the limbs. By the 13th day after the operation, the child was conscious with flexible eyes, and could understand words by blinking, but was still unable to speak. The right leg could move autonomously and bend slightly. Neck and limb muscle tone was slightly decreased, ankylosis was slightly alleviated, right wrist and feet flexed, and GCS was 8 points. Intermittent limb shaking times decreased, occurring 2−3 times per day on average.

**FIGURE 2:**
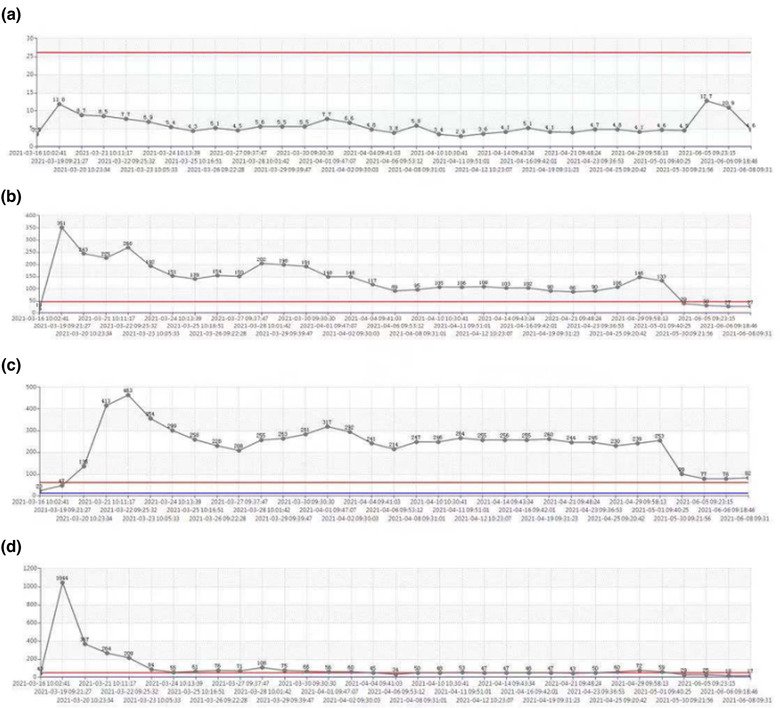
Postoperative liver function recovery trend line chart. (a) Bilirubin change trend chart. (b) Alanine aminotransferase change trend chart. (c) Aspartate aminotransferase change trend chart. (d) Glutamyl transpeptidase change trend chart

**FIGURE 3:**
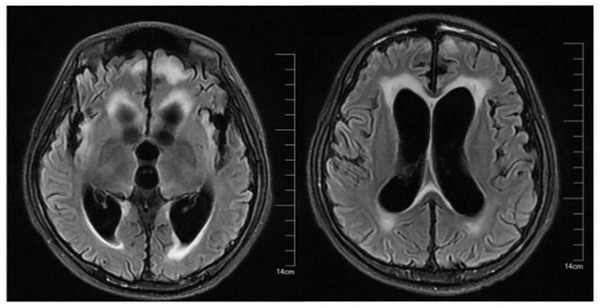
Postoperative cranial MRI

During postoperative treatment, the liver function of the child gradually recovered (Figure 2). The muscle strength and muscle tone of both lower limbs gradually recovered. The child was re‐examined regularly by cranial enhanced MRI, and hydrocephalus and ventriculomegaly showed no significant progress or improvement compared with preoperation (Figure 3). Encephalitis and other central nervous system lesions were ruled out by cerebrospinal fluid puncture. After consultation with neurology experts in other hospitals, internal and external pyramidal system injury was considered. On the 45th day after the operation, the child's lower limbs would move freely in bed, but the patient still could not stand. Muscle strength and muscle tone of both upper limbs were significantly relieved. Both hands could relax freely, and the upper limbs could be raised. The muscle tone of the right wrist was still slightly increased. GCS was 9 points. Considering the patient's recovery, slow nerve repair was considered. The patient was transferred to other hospitals for oxygen therapy and exercise of limb function.

**FIGURE 4:**
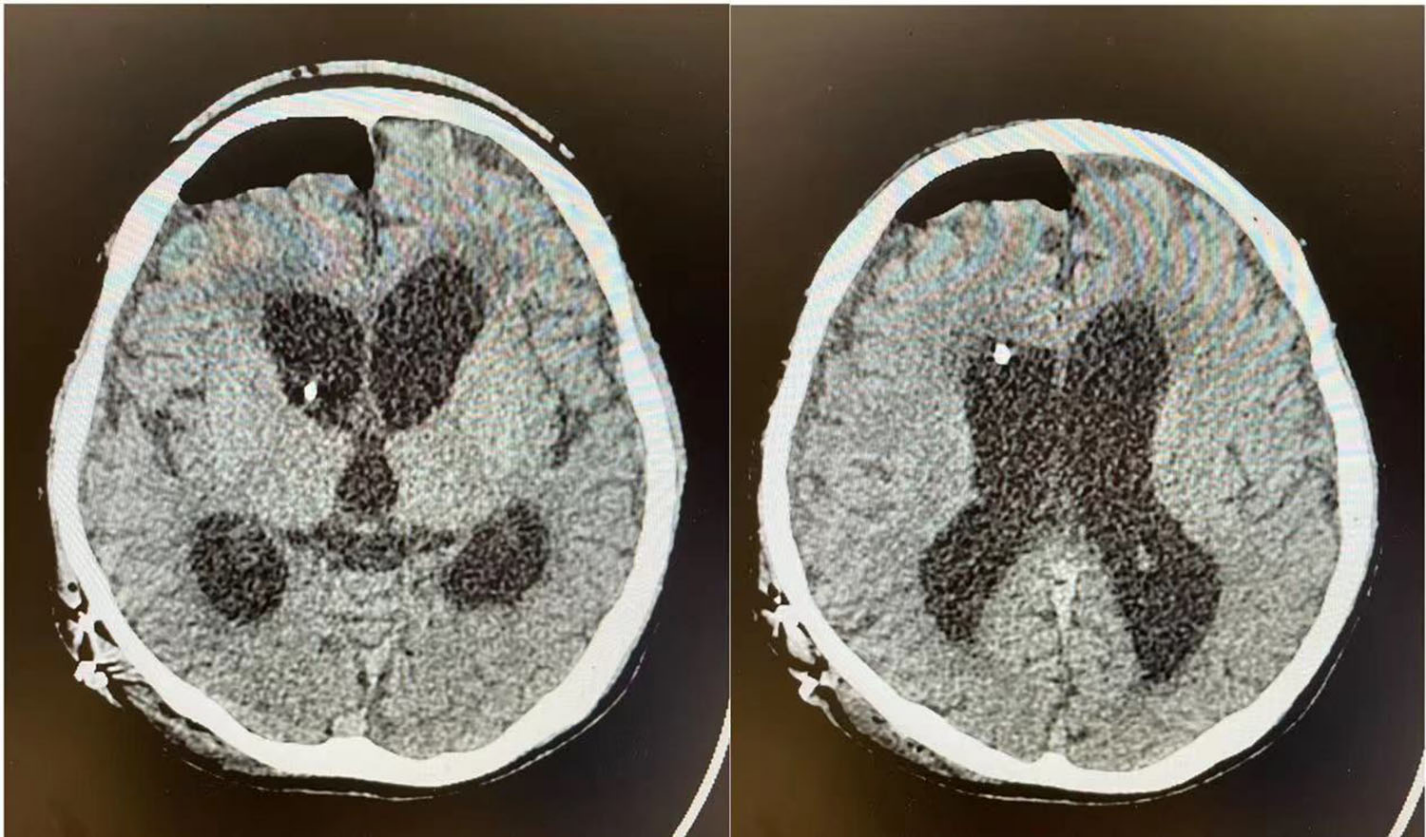
Postoperative head CT

Two months after LT, the child demonstrated unresponsiveness and incontinence. He came to the Department of Neurosurgery in our hospital. Head CT showed hydrocephalus and interstitial cerebral edema in the lateral ventricle. A lumbar puncture was performed. The pressure of cerebrospinal fluid was 120 mmH_2_O. We released 25 ml of cerebrospinal fluid, and the symptoms were relieved. After internal discussion in the department, the ventriculoperitoneal shunt was performed (June 3, 2021), and the pressure pump was adjusted to 90 mmH_2_O. Repeated head CT showed improvement of interstitial edema of the ventricle (Figure 4). And the ability to respond was improved, allowing the patient to be discharged.

Nine months after LT, the child was admitted to the hospital due to abnormal liver function. Head CT showed hydrocephalus and paraventricular interstitial cerebral edema were roughly the same as previously observed, while the bilateral frontal lobes and subcortical fissures showed patchy low‐density foci. The muscle strength of the limbs had returned to normal, and they could be moved freely. However, the child could not speak normally and could only pronounce single words and his intelligence level was low. But he can simply match graphics and words. GCS was 11 points. Based on the overall situation of the child, our diagnosis was that the child's body motor center had gradually returned to normal, but the language center had not improved.

## 3 DISCUSSION

Ammonia is a normal metabolite in the body, which mainly comes from the decomposition of amino acids, intestinal absorption, kidney generation, and other processes. Under normal physiological conditions, ammonia concentration can be maintained at a low level (< 60 μmol/L) through the urea cycle, glutamine synthesis, and kidney secretion. When blood ammonia is > 55 μmol/L (neonatal blood ammonia > 80 μmol/L), it can be diagnosed as hyperammonemia (Savy et al., 2018). The main clinical manifestations include vomiting, anorexia, mental and behavioral abnormalities, drowsiness, coma, and other symptoms. Genetic metabolic disease and liver function damage are the most common causes of hyperammonemia.

Under normal conditions, ammonia can freely diffuse across the blood–brain barrier and rapidly condenses with α‐ketoglutarate under the action of astrocyte glutamine synthase to form osmotic glutamine, which is the main pathway for ammonia detoxification in the nervous system. However, hyperammonemia has a strong toxic effect on the nervous system. On the one hand, large amounts of free ammonia penetrate the blood–brain barrier and are converted to glutamine in astrocytes. Glutamine is an osmotic agent. With the increase in intracellular concentration of glutamine, astrocytes gradually swell and are damaged, and eventually, brain edema or even brain hernia occurs (Summar & Mew, 2018). On the other hand, ammonia can also alter water transport through aquaporins, further aggravating astrocyte swelling and brain edema. Astrocyte swelling promotes calcium‐dependent glutamate release and inhibits glutamate‐aspartate transporter reuptake of glutamate. Studies have shown that when the brain is exposed to high concentrations of ammonia, the extracellular accumulation of glutamate promotes overexcitability of toxic cells by activating N‐methyl‐D‐aspartic acid (NMDA) receptors, leading to increased production of nitric oxide (NO), impaired mitochondrial respiration, ATP reserve depletion, free radical accumulation, and induced oxidative stress. This eventually leads to neuronal death (Auron & Brophy, 2012; Paprocka & Jamroz, 2012). Overactivation of NMDA receptors also activates neurotoxic pathways leading to axonal degeneration and cell death (Paprocka & Jamroz, 2012). Studies have shown that ammonia in stunted rats inhibits the growth of axons and dendrites and interferes with signal transduction pathways (Braissant et al., 2013). In addition, the large amount of α‐ketoglutarate consumption in the brain can lead to disorders of the tricarboxylic acid cycle, which affects the energy metabolism of the nervous system, and then causes drowsiness, irritability, disturbance of consciousness, convulsions, behavioral abnormalities, and other clinical manifestations. At the same time, because ammonia and potassium ions have similar ionic radii, ammonia can be transported by potassium ion carriers (such as the Na+/K+‐ATPase, Na+‐K+−2Cl‐ cotransporter) (Ott & Vilstrup, 2014). Therefore, the increase in blood ammonia will alter the balance of potassium ions in astrocytes, leading to an increase in extracellular potassium ion concentration, which affects inhibitory nerve conduction, thus causing neurological dysfunction and induction of encephalopathy (Rangroo Thrane et al., 2013). The above‐combined effects can lead to increased intracellular osmotic pressure and then cell edema, functional damage, and eventually brain edema and brain hernia (Summar & Mew, 2018). This irreversible damage to the brain can lead to seizures, cognitive impairment, cerebral palsy, and even death (Savy et al., 2018).

The primary way for the body to remove ammonia is through the urea cycle, which is mainly carried out in the liver (Choi et al., 2012; Tuchman & Plante, 1995). OTC is one of the key enzymes in the urea cycle. It is encoded by the OTC gene located on Xp21.1, and is mostly expressed in the liver and also in the mitochondrial matrix of the small intestine. Currently, a total of 503 mutations in the OTC gene have been identified (Shao et al., 2017). Mutation of the OTC gene can lead to a decrease or loss of OTC enzyme activity, which then results in disruption of citrulline synthesis and the ornithine cycle, blocking of ammonia degradation, and an increase of blood ammonia, causing OTCD (Smith et al., 2005). OTCD is an X‐linked genetic disorder in which only 15–20% of carriers have symptoms. It is believed that patients with the mutated OTC gene can develop hyperammonemia at any stage. Infection, high protein diet, fasting, steroid treatment, and other factors all have some influence on the onset time and disease course. At present, it is generally believed that environmental stress factors cause an increase in nitrogen catabolism load, which produces a negative nitrogen balance in patients and further diminishes already overburdened OTC activity, leading to hyperammonemia. The clinical manifestations, severity, and prognosis of OTCD are also closely related to the degree, speed, and duration of the elevation in blood ammonia. When blood ammonia is less than 100 μmol/L, most patients show no obvious symptoms. When blood ammonia reaches 100−200 μmol/L, excitement, abnormal behavior, vomiting, and anorexia tend to appear. At blood ammonia concentration more than 200 μmol/L, disturbance of consciousness and convulsions may occur. Finally, when blood ammonia reaches more than 400 μmol/L, coma, dyspnea, and even sudden death will likely follow.

At present, clinical tests for OTCD mainly include genetic tests and detection by blood tandem mass spectrometry and urine gas chromatography‐mass spectrometry. Genetic testing is the gold standard for the diagnosis of OTCD (Aiuti et al., 2007). The results of blood tandem mass spectrometry have shown that as glutamine increases and citrulline is decreased, glutamic acid and alanine increase, and arginine decreases. The main manifestation of urine gas chromatography‐mass spectrometry is an increase in urine lactate acid, which may be accompanied by an increase in uracil.

OTCD can also lead to brain imaging changes, which in most cases show different degrees of cerebral cortex swelling, brain edema, brain atrophy, and signal changes. In contrast, brain MRI in late‐onset OTCD shows cortical damage, including acute ischemia, ventriculomegaly, and defects in myelination. Therefore, the timing of MRI may be critical for finding these lesions. Takanashi et al. (2003) reported three cases of neonatal UCDs, and MRI examination of the three cases showed consistent specificity: the T1 phase presented large signal changes, while the T2 phase presented significant signal changes in the head of the caudate nucleus, middle of the putamen, insular lobe, and junction of the Rolandic region, whereas signal intensity was similar or reduced in the globus pallidus. He further grouped hyperammonemia encephalopathy by MRI, providing clues for early diagnosis (Takanashi et al., 2003). Bindu et al. (2009) reported MRI changes in three cases of acute hyperammonemia caused by different etiology. This study was similar to the previous report but was more extensive, showing diffuse cortical signal changes and whole‐brain swelling. Neuropathological studies have found that irreversible pathological lesions occur in UCD patients, including brain atrophy, ventricular enlargement, delayed myelination, cicatricial gyri formation, cortical spongiform degeneration, and the appearance of Alzheimer's type II astrocytes (Dolman & Clasen, 1988). In our case presentation, MRI changes in the patient were similar to those reported in the literature above. MRI images showed multiple abnormal signal shadows in the right frontal lobe, parietal lobe, and subcortical fissure of the lateral fissure. At the same time, there were manifestations of cerebral edema, including acute cerebral edema at the onset of the disease and interstitial cerebral edema after LT. Presently, a large number of studies have confirmed that hyperammonemia is closely related to cerebral edema (Bindu et al., 2009; Paprocka & Jamroz, 2012). In our case, the cerebral edema that developed at onset was likely due to the toxic effects of hyperammonemia on the nervous system. In contrast, interstitial cerebral edema after transplantation mostly occurs in the side of the ventricle, especially the side of the lateral ventricle, and is often accompanied by hydrocephalus, so it is also called hydrocephalus edema. This is due to the effect of hydrostatic pressure forcing some of the ventricular fluid to escape and seep into the adjacent white matter. The degree of edema is determined by the level of ventricular pressure. The repeated bouts of interstitial cerebral edema we observed in the child were likely due to persistent hydrocephalus.

The aforementioned MR findings are common imaging changes in patients with OTCD, while a rare finding of hydrocephalus was seen in our case. After reviewing a large amount of literature and excluding congenital developmental malformations in children, we speculate that hydrocephalus was related to the indirect effect of neurotoxicity caused by hyperammonemia. First, hyperammonemia can cause a neuroinflammatory response. In the early stage of inflammation, hydrocephalus is generated due to the malabsorption of diffuse cerebrospinal fluid (CSF), and in the later stage, it can lead to further obstruction of CSF circulation and a residual ependymal scar, resulting in intraventricular obstruction and multicompartment hydrocephalus (Andresen & Juhler, 2012). Second, the toxic effects of hyperammonemia can cause astroglial damage, as well as reactive gliosis. Xu et al. (2012) found that in a rat model of hydrocephalus, the degree of astroglial proliferation was positively correlated with the development of hydrocephalus. In addition, astrogliosis may lead to abnormal polarity and dysfunction of aquaporin‐4 in the lymphoid system (Back et al., 2017; Venkat et al., 2017), affecting the exchange of cerebrospinal and tissue fluid, which may further aggravate hydrocephalus. Finally, the exposure of the brain to high concentrations of ammonia results in increased production and extracellular accumulation of glutamate and NO (Rangroo Thrane et al., 2013; Shao et al., 2017; Tuchman & Plante, 1995), both of which play an important role in maintaining normal cerebral blood flow and neuroendocrine activity. However, under pathological conditions, excessively generated NO and glutamate have cytotoxic effects. In experimental hydrocephalus animals, NO and glutamate concentrations in the cerebrospinal fluid were positively correlated with the degree of ventricular compression, suggesting that NO and glutamate metabolism disorders may be involved in the development of hydrocephalus. However, there is no direct evidence showing that hyperammonemia is related to hydrocephalus in OTCD patients, so this relationship should be examined in future research. At present, improvement in imaging manifestations has not been observed in our patient, which is contradictory to clinical manifestations in other children; therefore, further observation, follow‐up, and discussion are needed.

At present, the treatment of OTCD is mainly based on long‐term dietary therapy, in which a low‐protein diet is the basis for treatment (Aiuti et al., 2007; Bloch et al., 2006). In addition, drugs, such as arginine, sodium benzoate, and sodium phenylbutyrate, can remove excess ammonia by creating a metabolic bypass. Acute hyperammonemia caused by coma requires immediate intravenous injection of blood‐lowering ammonia drugs, hemodialysis, or peritoneal dialysis to remove excessive blood ammonia as soon as possible. Previous studies (Enns, 2008; Tuchman et al., 2008) have confirmed the effectiveness of blood purification in rapidly reducing blood ammonia concentration. Although the severe hyperammonemia caused by genetic metabolic diseases can be controlled effectively by a low‐protein diet, drug therapy, blood purification, and other treatments, hyperammonemia is prone to recurrence. Therefore, LT can be considered in patients with congenital hyperammonemia when the condition is stable (McAllister, 2012). At present, there is no consensus on the timing of LT. In current clinical practice, the indications and timing of LT are mainly determined according to the clinical symptoms and disease progression of the patient, considering whether there is a drug‐resistant repeated increase in blood ammonia, obvious growth retardation, a decline in quality of life caused by strict dietary restrictions, as well as disease progression indicated by cranial MRI and other imaging examinations (Wakiya et al., 2011). Campeau et al. (2010) reported that early LT performed within 1 year after birth had a good prognosis. Kasahara et al. (2010) reported that in 124 OTCD children who received LT from November 2005 to May 2010, the 5‐year overall survival rate was 91.0%. Other research has shown that in the absence of dietary restrictions, 1‐ and 5‐year survival rates of children with hyperammonemia caused by UCDs after LT were 93.8% and 90%, respectively (Kasahara et al., 2010; Yamaguchi et al., 2006). However, there are still many controversies regarding the recovery of nervous system function after LT in children with OTCD. Some researchers have reported that the nervous system damage caused by hyperammonemia is irreversible, even if LT is performed, and that damaged nervous system function cannot be completely restored to normal after surgery (Wakiya et al., 2011; Wraith, 2001). In addition, long‐term diet control will lead to slower growth in children, which is also not conducive to the prognosis for LT (McDiarmid et al., 2004). However, it has also been reported that hyperammonemia in children can be corrected after LT, and the postoperative quality of life can be improved; furthermore, the neurological symptoms of some children have been improved, which was closely related to their preoperative status. Summar et al. (2013) considered that LTs in young children were high‐risk and required perioperative management. If the disease is stable and blood ammonia can be well controlled, then LT can be performed when the weight of the child has increased to 8 kg. LT should also be performed immediately in children with acute liver failure or with poor response to drug therapy for hypoglycemic ammonia (Wakiya et al., 2011). According to the case study we have provided, we believe that LT can fundamentally solve the problems of liver ammonia metabolism in OTCD patients with central nervous system injury, avoiding further damage to the central nervous system caused by hyperammonemia, and provide a basis for recovery of the central nervous system. At the same time, the nervous system of children has not yet been fully developed and is in the stage of neural development with great plasticity. Although the central nervous system is damaged, early LT is still possible for nerve repair.

OTCD causes about half of all congenital hyperammonemia. With the continuous development of surgical technology, we believe LT can not only thoroughly treat congenital hyperammonemia but also significantly improve the quality of life for patients, which will be conducive to their growth and development; in fact, LT has become the most effective means for the treatment of congenital hyperammonemia. Neurological damage caused by hyperammonemia has always been one of the key issues affecting the prognosis of patients with OTCD. The case we have provided has enabled an in‐depth discussion of this problem by presenting further clinical evidence documenting nerve injury and the possible repair of hyperammonemia caused by OTCD.

## COMPETING INTERESTS

The authors declare that they have no competing interests.

## AUTHOR CONTRIBUTIONS

All authors contributed to data analysis, drafting or revising the article, gave final approval of the version to be published, and agree to be accountable for all aspects of the work.

### PEER REVIEW

The peer review history for this article is available at https://publons.com/publon/10.1002/brb3.2765

## Data Availability

The references supporting the conclusions of this article are included within the article.
